# Functional Morphology of the Oral Jaws and Dentition Across Diverse Diets and Ontogeny in Prickleback Fishes (Stichaeidae)

**DOI:** 10.1002/jmor.70131

**Published:** 2026-05-12

**Authors:** R. C. Hoover, Joseph Heras, Kassandra L. Ford, Karly E. Cohen, Cassandra M. Donatelli

**Affiliations:** ^1^ University of Minnesota Twin Cities Minnesota USA; ^2^ Bell Museum of Natural History St. Paul Minnesota USA; ^3^ California State University San Bernardino California USA; ^4^ Friday Harbor Laboratories University of Washington Friday Harbor Washington USA; ^5^ University of Washington Tacoma Washington USA

**Keywords:** allometry, dentition, dietary diversity, fish feeding, functional morphology, jaws, MicroCT, ontogeny

## Abstract

Pricklebacks (Stichaeidae) are a useful model for studying dietary diversity in fishes. Several species integrate increasing amounts of plant material into their diets as they grow, which is matched by physiological adaptations and allometric growth of the digestive tract to enhance the digestion of plant material. Similar to physiological differences, there are varying mechanical demands of eating plants compared to a carnivorous diet; we hypothesize that oral jaw morphology, dental characteristics, and tooth functional variation reflect diverse diets in pricklebacks. To explore this relationship, we first captured high‐resolution jaw and dentition morphology using microCT scanning across four species of prickleback over ontogeny (*n* = 9 per species), and 3 outgroups (*n* = 3 per species). We compared traits between species and quantified the scaling of these traits over ontogeny. We then use linear discriminant analyses to identify traits that drive differences between dietary groups. Pricklebacks span a range of jaw and tooth morphotypes from large jaws with many villiform teeth, to short jaws with large, rounded teeth. While there is overlap between groups, we find several traits define dietary groups and diverge via allometry. However, comparison with outgroups reveals high morphological diversity within each dietary group.

## Graphical abstract

Prickleback fishes (Stichaeidae) feed upon diverse diets, spanning carnivory (orange), omnivory (blue), and herbivory (green). We used microCT scanning to explore the functional morphology of the oral jaws and dentition across species and ontogeny.

Focal species vary by morphotype and have divergent scaling patterns across diets. However, outgroups demonstrate high morphological diversity and show incongruence between feeding category and morphology.

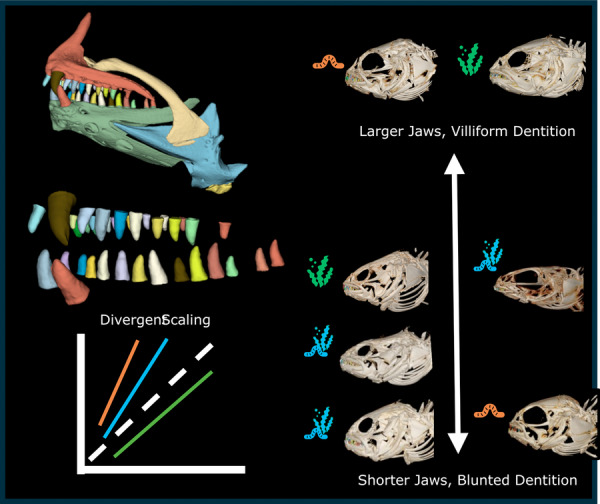

## 1 Introduction

### 1.1 Ontogeny Shapes Trophic Morphology

Teleost fishes often change what they eat, a pattern shaped by ecological opportunity, morphological constraint, and developmental changes (Davis et al. 2011; Sánchez‐Hernández et al. 2019; Stoner and Livingston 1984; Wainwright and Richard 1995). These dietary shifts may occur seasonally, as certain prey become more abundant; opportunistically, when prey become temporarily vulnerable; and ontogenetically, as growth and development alter what prey can be captured and processed. Ontogenetic diet shifts are virtually always present in fishes, with larvae and juveniles of the same species consuming markedly different prey; and subsequent shifts in prey type with growth are widespread (Davis et al. 2011; Eggold and Motta 1992; Mittelbach and Persson 1998; Piet 1998; Stoner and Livingston 1984; Wainwright and Richard 1995). Comparing how diverse species adapt to dietary changes over ontogeny can provide a nuanced understanding of how morphology evolves in response to dietary transitions (Davis et al. 2011; Piet 1998). Morphological scaling plays a key role in enabling these transitions. Isometric growth, such as increasing mouth gape with increasing body size, expands access to new prey, as seen in Caribbean serranids shifting from zooplankton to fishes (Wainwright and Richard 1995). In other species, dietary expansion involves allometric changes: an increase in jaw in‐lever in chimaera, for instance, results in a higher bite force, allowing them to process harder prey over ontogeny (Huber et al. 2008).

Tooth replacement further enhances dietary flexibility. All fishes are polyphyodont, continuously replacing their teeth (Berkovitz and Shellis 2023; Huysseune and Witten 2024). While this process helps maintain tooth function by replacing worn or damaged units, it also facilitates developmental changes in shape, number, and/or relative size of teeth (French et al. 2017; Huysseune and Witten 2024). In some fishes, ontogenetic shifts in dentition mirror changes in diet: simple conical teeth are replaced by bladed incisors for grazing in porgies (Stoner and Livingston 1984); sharp, multicuspid teeth emerge in Mexican tetras early in development for their transition from suction to biting (Trapani et al. 2005); and crushing molars develop in wolffish that specialize on hard‐shelled prey such as sea urchins (Bemis and Bemis 2015). In some Lake Victoria cichlids, the process of tooth replacement and regeneration has been shown to respond to changes in diet with plasticity in dental morphology (Gunter et al. 2013). Together, jaw morphology and dentition provide a functional and developmental record of trophic ecology across the life history of many fishes and stand as an integrative view of prey use over ontogeny.

### 1.2 Eating Plants Is a Challenge for Teleost Fishes

The ability to eat plants (i.e., omnivory and herbivory) has evolved at least 44 times across teleost fishes (Gerking 2014). However, there are multiple factors that restrict eating plants, and only 2%–5% of fishes are herbivorous (Choat and Clements 1998; Tolentino‐Pablico et al. 2008). Consuming plants virtually always requires an ontogenetic diet shift, where young fishes are carnivorous and plant matter is integrated with growth (Davis et al. 2011; Eggold and Motta 1992; Horn 1989; Stoner and Livingston 1984). While plants (such as grasses, leaves, algae, and seaweeds) may be more locally or seasonally abundant than animal prey, they are typically lower in calories, nitrogen, and phosphorus (Horn 1989; Horn and Neighbors 1984; Sanchez and Trexler 2016; Shantz et al. 2017; White 1985), all essential for fast‐growing juvenile fishes. Juvenile fishes are limited by the absorptive surface area in the gut against the relative volume of food necessary to make herbivory viable (German and Horn 2006). Higher plants may be more difficult to process due to indigestible cellulose, while red and brown algae contain different structural compounds such as carrageenan and alginate (Belovsky and Schmitz 1994; Montgomery and Gerking 1980; Steinberg 2024; Steinberg 1986). These digestive difficulties have been overcome through a variety of convergent adaptations across fishes, such as larger body cavities, increased absorptive surface area and longer food residence time within longer digestive tracts, (Burns 2021; Delariva and Neves 2020; German et al. 2010, 2014; German and Horn 2006; Huie et al. 2019), more muscular guts and crops to help breakdown prey (Arnette et al. 2024), changes to gut enzyme activities (Drewe et al. 2004; German et al. 2004; Heras et al. 2020; Herrera et al. 2022; Kim et al. 2014), and microbial supplementation or fermentation (German et al. 2015; Herrera et al. 2025).

Alongside shared adaptations in the digestive tract, we observe diverse jaw and dental adaptations for eating plants in omnivorous and herbivorous teleost fishes. These adaptations reflect the many different material properties and challenges associated with the vast number of plant genera and plant components consumed by fishes, from simple algae mats to the complex fruits and tough seeds of angiosperms (Hanley et al. 2007; Kolmann et al. 2024; Clements et al. 2009). While generally non‐evasive, many plants (such as macroalgae and aquatic vascular plants) differ in acquisition strategy from animal prey, often requiring the shearing or ripping of pieces from larger plants or the substrate rather than capture through suction or ram feeding (Buser et al. 2019; Norton 1995; Liem 1980). When biting as a mechanism for feeding upon fixed prey emerged in the Cenozoic, it allowed for access to new trophic niches such as herbivory and diversification in body shape in teleost reef fishes (Corn et al. 2022). Reef surgeonfishes possess incisiform teeth with quick moving jaws to precisely pull filamentous green algae (*Enteromorpha flexuosa*) from the substrate, while grazing cichlids in Lake Tanganyika possess strong jaws and bladed teeth to crop cyanobacteria from rocks (Purcell and Bellwood 1993; Tada et al. 2017; Yamaoka 1987). Across African cichlids from Lakes Malawi and Tanganyika, cranial morphology and jaw mobility are influenced by prey type, with herbivores demonstrating low kinesis compared to carnivores (Martinez et al. 2018). In South American pacus, adaptations to herbivory have led to distinct phenotypes, with some species possessing terminal jaws and robust teeth for crushing seeds, and others with subterminal jaws and slicing teeth for grazing upon riverweed (Podostemaceae) (Huie et al. 2019). In Australian grunters, herbivory has prompted both species diversification and evolution of novel tooth shapes (Davis et al. 2016). Exploring where and how herbivory has evolved across the diversity of fishes allows us to compare how both the digestive system and feeding morphology adapt under shared dietary pressures.

### 1.3 Dietary Diversity Across Ontogeny in Pricklebacks

One group of fishes that is a useful model for investigating how fishes adapt to dietary diversity, particularly through nutritional physiology, are the pricklebacks (Zoarcoidea: Stichaeidae). Pricklebacks are an enigmatic group of elongate marine fishes native to intertidal and subtidal habitats located in the temperate north Pacific (Eschmeyer and Herald 1983; Kells et al. 2016; Mecklenburg et al. 2002). Pricklebacks have been an accessible group for the study of elongate swimming, terrestrial locomotion, and lateral line function, but have received the most attention from dietary and nutritional physiologists (Clardy 2012; Clardy et al. 2015; Donatelli et al. 2017, 2021; Klein et al. 2013). The pricklebacks contain a diversity of diet types, with several instances of convergent evolution toward omnivory and herbivory; sympatric and closely related species with differing diets; and ontogenetic dietary shifts within species; all of which have positioned the pricklebacks as an attractive group for studying how fishes adapt to different trophic ecologies (Boyle and Horn 2006; German et al. 2014, 2015; German and Horn 2006; Heras et al. 2020; Herrera et al. 2025; Kim et al. 2014). Two species are fully herbivorous through convergent evolution and over ontogeny, *Xiphister mucosus* and *Cebidichthys violaceus*, feeding upon a wide range of marine algae genera such as *Ulva*, *Porphyra*, and *Gigartina* (Horn et al. 1982). These species digest algae with high efficiency, but with different strategies: *X. mucosus* relies on bulk intake, whereas *C. violaceus* utilizes microbial fermentation (German et al. 2015; Horn et al. 1986). *Xiphister atropurpureus*, sister species to *X. mucosus*, and *Phytichthys chirus*, sister species to *Xiphister*, integrate more algae into their diet with increasing size, making them ontogenetic omnivores (Gawlicka and Horn 2006; German et al. 2004, 2014; Kim et al. 2014; Rankins et al. 2023). In each of the species that consume plants, increases in the amount of algae consumed are matched by adaptations throughout the digestive system. Like many other groups of fishes, increased consumption of plant material is associated with positive allometry in digestive tract length over ontogeny, with herbivorous species having proportionally longer guts than omnivores (German et al. 2014; German and Horn 2006; Herrera et al. 2022; Rankins et al. 2023). The omnivorous *P. chirus* also has higher activity of chitinase, likely for digesting crustaceans (German et al. 2014, 2015; Rankins et al. 2023). These species have been contrasted with a consistent carnivore, the sympatric *Anoplarchus purpurescens*. *A. purpurescens* undergoes no ontogenetic changes in digestive morphology but does show increased expression of aminopeptidase, an enzyme important for digesting protein (German et al. 2014, 2015, 2016; German and Horn 2006). Additionally, *A. purpurescens* has an expansion of multiple protein digestion genes (i.e., trypsin and aminopeptidases) within its genome (Le et al. 2023). Pricklebacks primarily feed through biting, using their large oral jaws to grasp and apply force to prey items, as well as using spin feeding to rip pieces away from larger prey items like algae through rotation (Horn and Ojeda 1998; Miller and Marshall 1987; Yoshiyama et al. 1996).

Rich research in pricklebacks has identified a number of adaptations related to digesting plants. But how have diverse diets and ontogeny shaped the oral jaws and dentition in these species? In the pricklebacks, we expect the functional morphology of the oral jaws and dentition to reflect potentially disparate challenges presented by different prey across species and over ontogeny. We hypothesized that the functional morphology of the jaws and dentition would follow similar patterns to the digestive system, where ontogeny leads to the necessary specializations in fishes that consume plants. To answer these questions, we aimed to (1) describe and compare the morphology and ontogeny of the oral jaws and dentition across species and diverse diets, (2) explore functional differences between diet groups, and (3) compare our focal species to outgroups to test broader applicability across the superfamily Zoarcoidea.

## 2 Material and Methods

### 2.1 Specimen Selection and Micro‐CT Imaging

**Figure 1:**
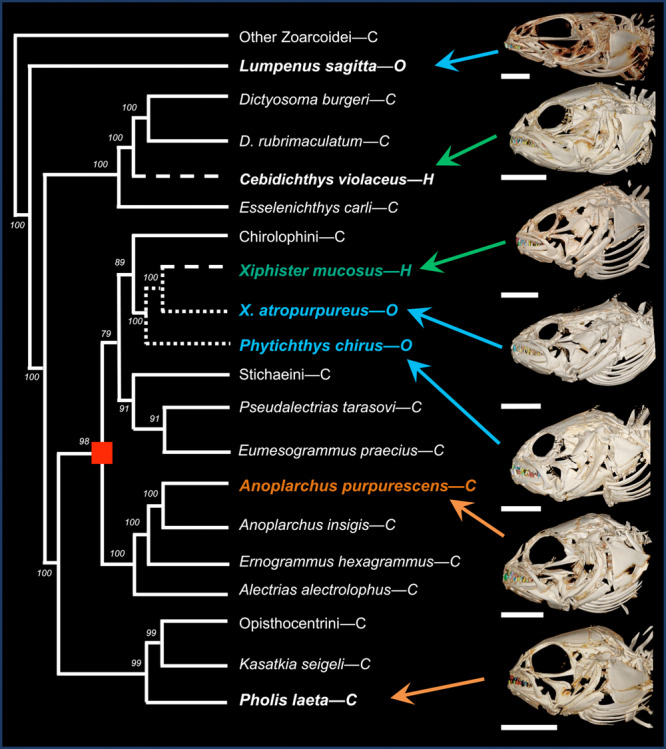
Skull Morphology and Diet across the Phylogenetic tree. Micro CT volume rendering of each species studied. Focal species in colourful text and the shared node for the focal clade distinguished by a red box. Phylogenetic hypothesis of Stichaeidae and related groups based on *cytb*, *16s*, and *tomo4c4* genes (Kim et al. 2014). Ontogenetic diet classes of H=herbivory, O=omnivory, C=carnivory. Predicted evolution of herbivory (dashed line) and omnivory (dotted) are shown. Bayesian posterior probabilities indicated on nodes. Individual scale bars = 5 mm.

We analyzed cranial morphology of four closely‐related species with well‐described diets over an ontogenetic size series. These species represent three dietary trajectories: *Xiphister mucosus* (herbivore, “H”), *Phytichthys chirus* and *Xiphister atropurpureus* (omnivores, “O”), and *Anoplarchus purpurescens* (carnivore, “C”), see Figure 1. We selected nine specimens from each species (sizes ranged from 67 to 179 mm total length; Table S1) to capture allometric changes in feeding morphology. We note that the sizes examined are after the initial ontogenetic diet shift in omnivorous and herbivorous pricklebacks (Barton 1982; Horn et al. 1982; Setran and Behrens 1993). To contextualise these patterns, we examined three sympatric outgroups with described diets in Zoarcoidea (*n* = 3 per species); *Cebidichthys violaceus*, an herbivore (German et al. 2004, 2015; German and Horn 2006; Wright et al. 2023), *Lumpenus sagitta*, an omnivore with carnivorous gut physiology (Rankins et al. 2023), and *Pholis laeta*, a carnivore (Hughes 1985; Peden and Hughes 1984). We limit the scope of our analyses to the oral jaws and dentition to focus on differences in prey acquisition, but acknowledge that pharyngeal jaw morphology is likely an important factor for prey processing and feeding ecology as a whole (Lauder 1983; Wainwright 2005).

We acquired 40 ethanol‐preserved specimens from the Burke Museum of Natural History and Culture (UWFC) and the Friday Harbor Laboratories (FHL) collections. We also borrowed three preserved *L. sagitta* specimens from the research collections of J. Heras. Lastly, we collected two additional fishes from tidepools at FHL. These fish were maintained in a flow‐through seawater system, then euthanized with an overdose of MS‐222. All animals were collected and housed under IACUC protocol #4238‐03. We fixed the specimens with buffered formalin and preserved them in 70% ethanol. We micro‐CT scanned specimens with a Bruker 1173 SkyScan at the Karel F. Liem Bio‐imaging Facility at FHL. Micro‐CT allows for nondestructive imaging, important for the use of museum specimens (Ford et al. 2023). Micro‐CT produces 3D models that can be easily processed digitally using open source software, and preserves data such as volume that cannot be easily estimated using traditional stereomicroscopy. However, we acknowledge that other methods such as scanning electron microscopy may reveal fine cusp morphology or microscopic tooth wear patterns that are not captured here. All samples were scanned between 7.4 and 18.1 μm, with an exposure ranging from 1100 to 1180 ms, a voltage of 65 kv, and an amperage of 123 μA (Table S1). Voxel size was adjusted based on relative specimen size in order to balance relative scan resolution and file size. We reconstructed scans using Bruker *NRecon* software (Bruker microCT, Kontich, Belgium 2016). All reconstructed scans are freely available through Morphosource.org https://www.morphosource.org/projects/000731353 (Boyer et al. 2016).

### 2.2 Jaw Morphometrics, Dental Characteristics, and Functional Homodonty Modeling

**Figure 2:**
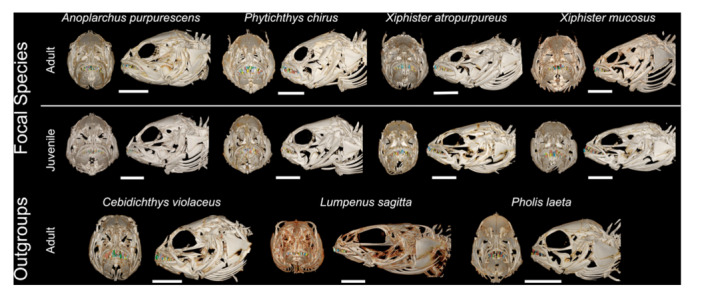
Micro CT volume renderings of skull morphology. Dentition segmentations overlaid, multicoloured. Adult fish scale bars = 5 mm, juvenile = 2.5 mm.

We imported reconstructed imagestacks into 3D‐Slicer using the *Slicermorph* extension (version: d9b1c22, Kikinis et al. 2014; Pieper et al. 2004; Rolfe et al. 2021). We generated volume renderings of each skull and used the Markups module to measure nine linear morphometrics of the skull and the left side of the oral jaws, following techniques from Buser et al. (2020; Figure 2 and Table 1). The relative size and proportions of the oral jaws dictate the velocity and force of jaw closing (Westneat 1994), and can reveal how these fishes acquire prey, such as fast opening jaws for rapid suction, or shorter jaws for more forceful bites (Buser et al. 2019; Liem 1980; Norton 1995; Westneat 2004). We based our nine linear morphometrics on lever mechanics and joint‐linkage models, useful simplifications for the complex musculoskeletal system of craniofacial bones in fishes (Barel 1982; Hulsey and García De León 2005; Wainwright and Richard 1995; Westneat 1990, 2004). We focused on two joints within the craniofacial system, the lower jaw joint of the articular‐quadrate and the upper jaw joint of the maxilla‐palatine. We measured premaxilla ascending process height and the opening in‐lever of the lower jaw, measures of jaw protrusion and speed of jaw opening (Bellwood et al. 2015; Motta 1984; Westneat 2004), closing in‐lever of the lower jaw and upper jaw (maxilla length), measures of relative closing force (Wainwright and Richard 1995; Westneat 2004), out‐lever for the lower and upper jaws, the distance between the jaw joint and the anterior edge of each jaw, premaxilla length, and horizontal jaw width, a predictor of maximum prey size (Heiple et al. 2023; Mihalitsis and Bellwood 2017).

**Table 1:** Jaw morphometrics descriptions.

Number	Measurement	Description
1	Skull length	length from the anterior tip of vomer to the posterior edge of the basioccipital
2	Jaw width	distance between lower jaw joints
3	LJ out‐lever	length from anterior most medial point of dentary to lower jaw joint
4	LJ closing in‐lever	length from doral most point of the ang‐articular to lower jaw joint
5	LJ opening in‐lever	length from medial most point of the retro‐articular to lower jaw joint
6	Premaxilla height	length from anterior edge of premaxilla to dorsal most tip of ascending process
7	Premaxilla length	length from anterior edge of premaxilla to the posterior most edge
8	UJ out‐lever	length from anterior edge of premaxilla to dorsal most part of upper jaw joint
9	UJ closing in‐lever	length of the maxilla from posterior most edge to dorsal most part of upper jaw joint

**Figure 3:**
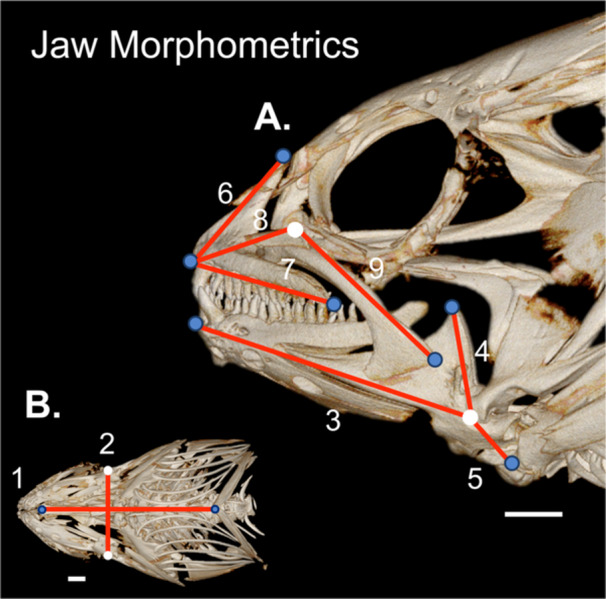
Jaw morphometric scheme (*X. mucosus*). (A) Lateral view. (B) Ventral View. Measurements described in Table 1. Blue circles are homologous landmarks. White circles are the jaw joint for the upper and lower jaw respectively. Red lines represent linear measurements. Scale bars = 1 mm.

After linear morphometrics, we generated a segmentation of the jaws and used the Scissor and Island tools to create individual segments for every tooth of the upper and lower jaws (premaxilla and dentary). We found the vast majority of teeth to be well mineralized and attached to the jawbones, and teeth undergoing replacement were not used because they were incompletely mineralized. We then used the *Dental Dynamics* (version: d4875f0, Cohen et al. 2024) module as part of the *SlicerBiomech* extension to automatically calculate dental characteristics (height, width, and surface area) and biomechanics (leverage, stress) for every tooth. We assumed simple lever mechanics (Figure 3), with a single muscle insertion point for the in‐lever of the lower jaws (dorsal‐most point of the angulo‐articular) and upper jaw (posterior point of the maxilla). Since jaw muscle composition was not visible in our CT‐scans, we used a static bite force of 1 Newton and a muscle insertion angle of 90 degrees in *Dental Dynamics*, mirroring relative mechanical advantage for each tooth.

We processed data from *Dental Dynamics* using the functional homodonty method described in Cohen et al. (2020a, 2020b), adapting their publicly available R code to fit our dataset. We used the functional homodonty method to identify individual teeth that function differently than the rest of the dentition (i.e., functionally heterodont teeth), based on their stress relative to the rest of the dentition. To make the evolutionary and functional diversity of dentitions more testable, anatomists often group dentitions into two categories: *morphologically homodont*, where all teeth are similar in shape and/or size, and *morphologically heterodont*, where teeth within the same dentition differ in shape and/or size (Conway et al. 2015; Huie et al. 2020; Schwartz 1982). However, these categories leave out important variation that affects dental function; even morphologically similar teeth can have functional differences based on relative size and position within a given jaw. For example, large canines can change in function from primarily prey holding when located at the front of the jaw to prey fracture and processing near the rear of the jaw due to increased leverage (Cohen et al. 2020a; Cohen et al. 2020b; Mihalitsis and Bellwood 2019). These teeth, though morphologically homodont, can be categorized as “functionally heterodont”. *Functionally homodont* teeth have similar stress values to the rest of the dentition, while *functionally heterodont* teeth have stress values that fall outside of the typical range, with either much higher or lower stress values than the rest of the dentition. Different diets may require different frequencies and distributions of functionally heterodont teeth. This method is thoroughly described by Cohen et al. (2020a), but in brief: We normalized stress values for each dentition by the median stress of each jaw. In order to generate a threshold for significant differences in function, we first performed a bootstrapping analysis as outlined in Cohen et al. (2020b). We randomly subsampled half of each dentition without replacement and normalized by the subsampled median stress. We repeated this 10,000 times for each dentition to create a null distribution of residual stress values. We then used a k‐means cluster analysis to identify clusters of stress values within our data set (*n* = 2) and generated a threshold for homodonty as the mean of the two clusters. Teeth within our generated threshold were considered functionally homodont, while teeth that fell outside of this threshold were functionally heterodont (Figure S1 and S2). From this analysis we generated two summary values for each dentition: (1) proportion of heterodont teeth and (2) average squared residual stress (Table S4). The proportion of heterodont teeth infers how many teeth were functionally different while averaged squared residual stress refers to the magnitude of divergence across the dentition.

### 2.3 Statistical Analysis

All statistical analyses were performed in the *Posit/RStudio* environment version 4.4.1 (R Core Team 2024) and plotted using *ggplot2* version 3.5.0 (Wickham and Sievert 2009).

We used the *stats* package to perform non‐parametric Kruskal–Wallis tests to determine differences for both jaw morphometrics and dental characteristics across species (R Core Team 2024). If Kruskal–Wallis tests were significant, we performed post hoc pairwise wilcoxon tests, with a false discovery rate correction of 0.05, to identify which specific species were significantly different (Table 3). Jaw morphometrics and dental characteristics were size‐corrected over skull length.

**Figure 4:**
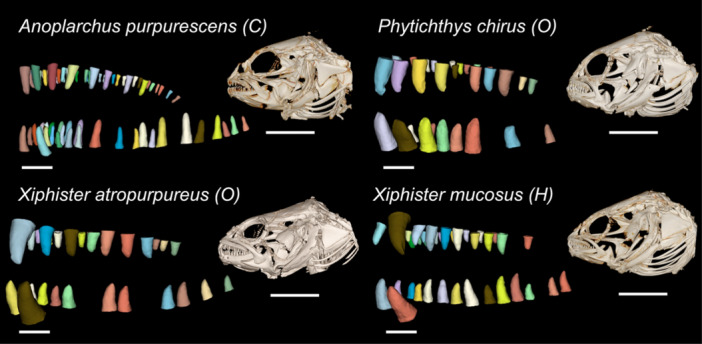
Dentition Segmentations for Focal Species. Each tooth is an individual segmentation, colourized, dentition scale bar = 0.5 mm. Specimen near mean for ontogenetic size range. Representative volume rendering of skull morphologies, scale bar = 5 mm.

We used the *caret* and *MASS* packages to perform a series of three linear discriminant analyses to investigate which traits (jaw morphometrics, dental characteristics, and functional heterodonty) contributed the most to differences between diets (Figure 4) (version 7.0 and version 7.3; Kuhn 2008; Venables and Ripley 2013). Linear discriminant analyses reduce dimensionality by maximizing distance between assigned classes on reduced axes (Engelman 2000). Each species was grouped into classes of carnivorous, omnivorous, or herbivorous based on established dietary trends (German et al. 2004, 2014; German and Horn 2006). We used a series of trait combinations to explore the usefulness of the different types of traits for increasing separation between dietary groups.

**Figure 5:**
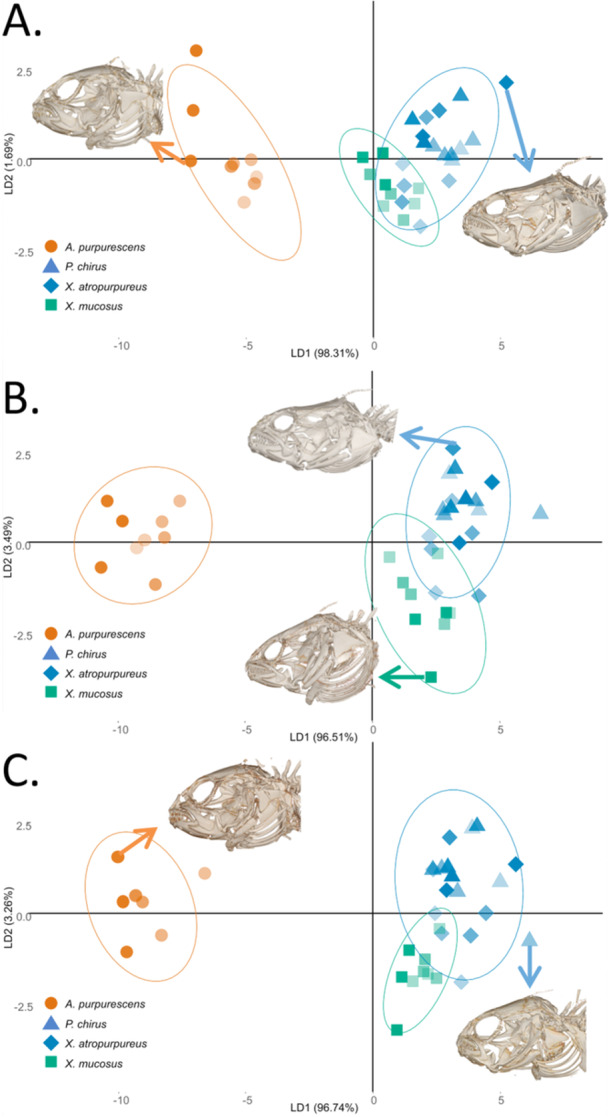
Linear Discriminant Analyses for Diet Classes. (A) Jaw morphometrics. (B) Jaw morphometrics and dental characteristics. (C) Jaw Morphometrics, dental characteristics, and proportion of functionally heterodont teeth (Table 4). Ellipses indicated 95% confidence intervals using a multivariate *t*‐distribution. Orange ellipse (*Anoplarchus purpurescens*) indicates carnivory, green ellipse (*Xiphister mucosus*) indicates ontogenetic herbivory, and blue ellipse (*X. atropurpureus* and *Phytichthys chirus*) indicate ontogenetic omnivory. Opacity scaled by relative size (larger skulls = darker alpha). Skulls shown have the largest absolute value (data point shown by arrows) for LD1 imaged for LDAs A and C, in LDA B skulls have the largest absolute values across LD2.

**Figure 6:**
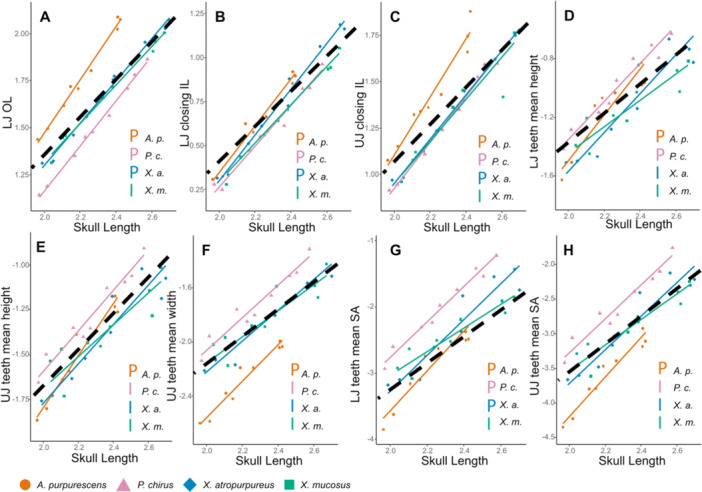
Reduced‐major axis regressions of top 8 LDA loadings and skull length. (A) lower jaw out‐lever, (B) lower jaw closing in‐lever, (C) upper jaw closing in‐lever, (D) lower jaw teeth mean height, (E) upper jaw teeth mean height, (F) upper jaw teeth mean width, (G) lower jaw teeth mean surface area, (H) upper jaw teeth mean surface area. Axes are log scaled. Dashed lines represent theoretical isometric slopes. Letters denote scaling patterns of each species: P = positive allometry, I = isometric growth. Abbreviations: LJ = lower jaw, UJ = upper Jaw, IL = in‐lever, OL = out‐lever, SA = surface area. P = positively allometric growth, I = isometric growth. Top eight traits allometric tests in Table 5, expanded allometric tests are in Table S2.

We used reduced major‐axis regressions in the *Lmodel2* package (version 1.7, LaBarbera 1989; Legendre and Oksanen 2018) to examine how jaw morphometrics and dental characteristics scale over ontogeny within the four focal species. We first calculated slopes for each trait based on relative skull length, then rejected isometry if the theoretical isometric slope fell outside of a 95% confidence interval of the calculated slope. All scaling data were log‐transformed. We visualized the ontogenetic regressions for the top eight Linear Discriminant Analysis (LDA) loadings from our LDA analyses (Figure 5 and Table 4) in Figure 6. We provide the regression for these traits in Table 5, and expand to all traits in Table S2.

## 3 Results

### 3.1 Feeding Morphology of Pricklebacks

When examining the morphometrics of the oral jaws, we found significant differences across species. Within the focal clade, carnivorous *A. purpurescens* is the most different from the other three species. *A. purpurescens* and *P. chirus* are the most similar, but possess significant differences in relative means for six of eight traits (Figures 2 and 4, Tables 2 and 3). They do not differ in relative opening in‐lever of the lower jaw and the height of the premaxilla ascending process, both of which are proxies of jaw protrusion capability (Table 3). *A. purpurescens* differs from both *Xiphister* species for all jaw measurements except the closing in‐lever of the lower jaw (more similar to *X. atropurpureus*). When compared to the outgroup species, *A. purpurescens* is the most similar to *C. violaceus*, with similar relative jaw widths, lower jaw closing and opening in‐levers, and premaxilla lengths, but *A. purpurescens* possess a longer lower jaw length, shorter premaxilla height, and longer upper jaw in‐lever and upper jaw length. *A. purpurescens* differs in most jaw morphometrics from the other outgroups, *P. laeta* and *L. sagitta*.

**Table 2:** Means and Standard Error.

Trait	Ap mean	Ap SE	Pc mean	Pc SE	Xa mean	Xa SE	Xm mean	Xm SE	Cv mean	Cv SE	Pl mean	Pl SE	Ls mean	Ls SE
Jaw Width	0.536	0.022100	0.345	0.008210	0.302	0.020100	0.373	0.032400	0.408	0.025800	0.261	0.019700	0.315	0.009560
LJ out‐lever	0.645	0.016200	0.458	0.006730	0.518	0.007040	0.508	0.003800	0.586	0.001190	0.468	0.015700	0.488	0.001890
LJ closing in‐lever	0.205	0.004500	0.185	0.002940	0.203	0.005900	0.188	0.002990	0.198	0.001540	0.169	0.007340	0.155	0.002700
LJ opening in‐lever	0.122	0.003810	0.110	0.002370	0.107	0.002530	0.106	0.002680	0.131	0.003020	0.099	0.004560	0.080	0.001070
Premaxilla height	0.224	0.006160	0.217	0.003640	0.203	0.002730	0.205	0.002320	0.269	0.003380	0.216	0.007200	0.211	0.001810
Premaxilla length	0.371	0.012300	0.292	0.006270	0.287	0.018200	0.290	0.019500	0.352	0.002360	0.244	0.009730	0.291	0.004750
UJ closing in‐lever	0.463	0.017700	0.365	0.006860	0.375	0.006560	0.367	0.010400	0.388	0.005000	0.284	0.015700	0.304	0.007210
UJ out‐lever	0.184	0.003660	0.138	0.001700	0.128	0.004170	0.137	0.015600	0.143	0.006140	0.144	0.006130	0.191	0.003640
LJ teeth mean height	0.034	0.001400	0.038	0.001040	0.032	0.001570	0.031	0.001010	0.034	0.001820	0.033	0.002300	0.028	0.001220
UJ teeth mean height	0.025	0.000753	0.029	0.000553	0.024	0.000804	0.024	0.000899	0.023	0.000664	0.027	0.001920	0.017	0.000451
LJ teeth mean width	0.016	0.000591	0.025	0.000653	0.020	0.000905	0.019	0.000491	0.017	0.001300	0.019	0.001770	0.014	0.000870
UJ teeth mean width	0.011	0.000287	0.018	0.000442	0.016	0.000480	0.016	0.000453	0.012	0.000590	0.016	0.001390	0.008	0.000415
LJ teeth mean SA	0.0007	0.000045	0.0014	0.000077	0.0009	0.000087	0.0008	0.000047	0.0007	0.000089	0.0008	0.000154	0.0005	0.000034
UJ teeth mean SA	0.0003	0.000019	0.0008	0.000037	0.0005	0.000040	0.0005	0.000029	0.0004	0.000027	0.0006	0.000109	0.0002	0.000019
LJ teeth mean number	25.500	1.550000	11.100	0.217000	14.600	1.020000	17.200	1.070000	28.200	0.441000	8.000	1.040000	10.200	0.167000
UJ teeth mean number	39.900	3.100000	20.700	1.690000	24.100	1.160000	24.200	1.890000	42.300	6.130000	12.000	1.730000	55.800	3.380000

Abbreviations: Ap = *Anoplarchus purpurescens*, Cv = *Cebidichthys violaceus*, Ls = *Lumpenus sagitta,* Pc = *Phytichthys chirus*, Pl = *Pholis laeta*, Xa = *Xiphister atropurpureus*, Xm = *Xiphister mucosus*.

**Table 3:** Comparison of means (Kruskal–Wallis, pairwise Wilcoxon Rank Sum Test).

Trait	df	*χ*	*p*	Ap ‐ Pc	Ap ‐ Xa	Ap ‐ Xm	Ap ‐ CV	Ap ‐ Pl	Ap ‐ Ls	Pc ‐ Xa	Pc ‐ Xm	Pc ‐ Cv	Pc ‐ Pl	Pc ‐ Ls	Xa ‐ Xm	Xa ‐ Cv	Xa ‐ Pl	Xa ‐ Ls	Xm ‐ Cv	Xm ‐ Pl	Xm ‐ Ls	Cv ‐ Ls	Cv ‐ Pl	Ls ‐ Pl
Jaw Width	6	28.73	**< 0.001**	**< 0.001**	**< 0.001**	**< 0.01**	0.055	**0.032**	**0.032**	0.149	0.635	0.076	**0.032**	0.076	0.149	0.076	0.562	1.000	0.635	0.121	0.258	0.149	0.149	0.149
LJ out‐lever	6	37.51	**< 0.001**	**< 0.001**	**< 0.001**	**< 0.001**	**0.017**	**0.017**	**0.017**	**< 0.001**	**< 0.001**	**0.017**	0.506	0.084	0.482	**0.017**	0.059	0.084	**0.017**	0.059	0.084	0.117	0.117	0.700
LJ closing in‐lever	6	24.59	**< 0.001**	**0.038**	0.836	**0.039**	0.329	**0.039**	**0.038**	**0.039**	0.541	0.069	0.124	**0.038**	0.070	1.000	**0.039**	**0.038**	0.124	**0.039**	**0.038**	0.124	0.124	0.124
LJ opening in‐lever	6	24.1	**<;0.001**	0.076	**0.024**	**0.025**	0.435	**0.038**	**0.024**	0.541	0.767	**0.024**	0.140	**0.024**	0.931	**0.024**	0.180	**0.024**	**0.024**	0.180	**0.024**	0.140	0.140	0.140
Premaxilla height	6	21.13	**< 0.01**	0.507	**0.032**	**0.032**	**0.032**	0.668	0.235	**0.043**	0.105	**0.032**	1.000	0.595	0.668	**0.032**	0.293	0.235	**0.032**	0.293	0.235	0.210	0.210	0.735
Premaxilla length	6	28.75	**< 0.001**	**< 0.001**	**< 0.001**	**< 0.01**	0.764	**0.024**	**0.024**	0.605	0.541	**0.024**	**0.042**	0.700	0.931	**0.024**	0.121	0.764	**0.024**	0.121	0.605	0.150	0.150	0.150
UJ closing in‐lever	6	31.16	**< 0.001**	**< 0.001**	**< 0.001**	**< 0.001**	**0.019**	**0.019**	**0.019**	0.509	0.573	0.204	**0.019**	**0.019**	0.796	0.533	**0.019**	**0.019**	0.370	**0.035**	0.064	0.150	0.150	0.494
UJ out‐lever	6	28.64	**< 0.001**	**< 0.001**	**< 0.001**	**0.02**	**0.024**	**0.024**	0.460	0.111	**0.021**	0.562	0.635	**0.024**	0.635	0.131	0.131	**0.024**	0.111	0.111	0.111	0.131	1.000	0.131
LJ teeth mean height	6	18.39	**< 0.01**	0.217	0.507	0.210	0.955	0.898	0.191	0.059	**< 0.01**	0.210	0.255	**0.064**	0.978	0.507	0.955	0.210	0.507	0.507	0.210	0.210	1.000	0.210
UJ teeth mean height	6	23.11	**< 0.001**	**< 0.01**	0.541	0.737	0.737	0.737	**0.024**	**< 0.01**	**< 0.01**	**0.024**	0.455	**0.024**	0.737	0.864	0.175	**0.024**	0.764	0.541	**0.024**	0.175	0.175	0.175
LJ teeth mean width	6	33.39	**< 0.001**	**< 0.001**	**< 0.01**	**< 0.01**	0.907	0.204	0.150	**< 0.01**	**< 0.001**	**0.021**	**0.038**	**0.021**	0.807	0.111	0.807	**0.021**	0.111	1.000	**0.021**	0.494	0.262	0.150
UJ teeth mean width	6	35.82	**< 0.001**	**< 0.001**	**< 0.001**	**< 0.001**	0.435	**0.015**	**0.015**	**< 0.01**	**< 0.01**	**0.015**	0.180	**0.015**	0.864	**0.015**	0.864	**0.015**	**0.015**	0.864	**0.015**	0.131	0.131	0.131
LJ teeth mean SA	6	28.48	**< 0.001**	**< 0.001**	**0.044**	0.051	0.907	0.489	0.162	**0.013**	**< 0.001**	**0.027**	**0.044**	**0.027**	0.804	0.293	0.804	**0.027**	0.293	1.000	**0.027**	0.162	0.804	0.162
UJ teeth mean SA	6	35.62	**< 0.001**	**< 0.001**	**< 0.001**	**< 0.01**	0.562	**0.029**	**0.017**	**< 0.01**	**< 0.001**	**0.017**	0.096	**0.017**	0.737	**0.017**	0.907	**0.017**	**0.029**	1.000	**0.017**	0.124	0.124	0.124
LJ teeth mean number	6	36.03	**< 0.001**	**< 0.01**	**< 0.01**	**0.010**	0.265	**0.031**	**0.031**	**0.039**	**0.008**	**0.031**	**0.032**	0.064	0.192	**0.031**	**0.039**	0.083	**0.031**	**0.031**	**0.031**	0.095	0.117	0.181
UJ teeth mean number	6	30.53	**< 0.001**	**0.028**	**0.028**	**0.033**	0.764	**0.034**	**0.055**	0.160	0.155	**0.039**	0.053	**0.034**	0.859	**0.034**	**0.034**	**0.034**	**0.046**	**0.034**	**0.034**	0.124	0.124	0.124

*Note:* Significant *p* values are bolded.

Abbreviations: Ap = *Anoplarchus purpurescens*, Cv = *Cebidichthys violaceus*, Ls = *Lumpenus sagitta,* Pc = *Phytichthys chirus*, Pl = *Pholis laeta*, Xa = *Xiphister atropurpureus*, Xm = *Xiphister mucosus*.

The other species from the focal clade, *P. chirus* and both *Xiphister* species, have broad overlap in most jaw morphometrics, but *P. chirus* possesses shorter lower jaws, taller premaxilla ascending process than *X. atropurpureus*, and longer upper jaw length than *X. mucosus*. No jaw morphometrics are significantly different between the two *Xiphister* species. When compared to outgroup taxa, *P. chirus* and both *Xiphister* are most similar to *P. laeta*. All three species differ from *C. violaceus* in the length of the lower jaw, the lower jaw opening in‐lever, and premaxilla height and length. Similarly, all three species differ from *L. sagitta* in the rear of the lower jaw through the closing and opening in‐lever, and *P. chirus* and *X. atropurpureus* also differ from *L. sagitta* in the upper closing in‐lever and out‐lever. We found no support for pairwise differences in jaw morphometrics between outgroup species, likely due to low sample size.

Morphology and pattern of dentition within the jaw varied across species (Figures 2 and 4, Tables 2 and 3). While all species have simple teeth without identifiable blades or cusps, their dentitions vary between numerous thin teeth (villiform dentition) to few, relatively large teeth (macrodont dentition) (Figure 4), a common axis of functional variation (Crofts et al. 2020; Freeman and Lemen 2007; Gidmark et al. 2019; Humphreys 1948; Lucas 2004; Mihalitsis and Bellwood 2019). *A. purpurescens* possesses large, villiform teeth. In the upper jaw, teeth are arranged with roughly evenly‐spaced large teeth on the lateral margin and smaller teeth medially. They lack anterior caniniform teeth, and the lower jaw has a similar gradient of small teeth punctuated by larger teeth. The two *Xiphister* species have a macrodont dentition with simple conical teeth that are slightly recurved in the upper and lower jaws. The lower jaw has a single row of large teeth with 1–2 large caniniform teeth at the anterior‐most position, and a cluster of small teeth located medially. The upper jaw has a similar pattern, but with a higher number of medially located small teeth, and these teeth continue further posteriorly. *P. chirus* has even larger teeth that are much more blunted in shape than *Xiphister* species. *P. chirus* has a similar pattern of dentition as the *Xiphister* species but lacks the largest teeth positioned in the anterior upper jaw, leading to a distinct underbite. Our three outgroup species span three distinct phenotypes (Figures 1 and 3, Table S3). *C. violaceus* has large jaws and villiform dentition similar to *A. purpurescens*. *P. laeta* has a dentition similar to *P. chirus*, with blunted teeth that are far fewer in number. Lastly, *L. sagitta* has an upper jaw of villiform teeth similar to *A. purpurescens* and *C. violaceus*, but with far less teeth in the lower jaw.

Similar to jaw morphometrics, we found support for species differences in dental characteristics across species (Tables 2 and 3). *A. purpurescens* possesses fundamentally different dental characteristics from our other focal species, differing from *P. chirus* in almost every trait and *Xiphister* in most traits except tooth height and the mean tooth surface area in the lower jaw for *X. mucosus*. *A. purpurescens* has no differences in dental characteristics from *C. violaceus*, but differs from *P. laeta* and *L. sagitta* in several ways such as number of teeth, upper jaw tooth width and surface area.

Our next focal species, *P. chirus*, has much larger teeth than both *Xiphister* species, reflected by significant differences in most dental characteristics. *P. chirus* also has fewer teeth in the lower jaw, but not the upper jaw. The larger and fewer number of teeth also distinguish it from *C. violaceus* and *L. sagitta*. *P. laeta* has the most similar teeth to *P. chirus*, differing in lower jaw mean tooth width, surface area, and number. No dental characteristics are significantly different between the two *Xiphister* species, and they differ from outgroup species in similar patterns. Both species are most similar to *P. laeta*, with the only differences being higher tooth number for both jaws; they are moderately similar to *C. violaceus* with significant differences in the width and surface of the teeth in the upper jaw, and differ from *L. sagitta* in almost every trait.

To compare functional variation within dentitions, we calculated whether each tooth is functional homodont or heterodont. We generated a cutoff for functional homodonty (± 0.963), where teeth with an absolute residual stress outside of that value are considered functionally heterodont. We then calculated the proportion of functionally heterodont teeth for each fish. We also calculated average squared residuals for each dentition, a measure of the magnitude in variability within dentitions.

In the lower jaw, we observe some individuals with functionally heterodont teeth within each species, but no clear ontogenetic trends (Figure S1 and Table S4). These functionally heterodont teeth are located either close to the posterior jaw joint or at the anterior of the lower jaw. We find similar patterns of heterodonty across our focal species, but also identify some species‐specific differences. In *A. purpurescens*, the mean proportion of heterodont teeth is 2% for the species. Heterodonty is more common in *P. chirus*, *X. atropurpureus*, and *X. mucosus*. The mean proportion of functionally heterodont teeth is highest in *P. chirus*, with a mean of 17.8% and every specimen possessing at least one heterodont tooth, followed by *X. atropurpureus*, with a mean of 11%. *X. mucosus* has the second lowest mean proportion of functionally heterodont teeth at 6.7%. In *P. chirus* and the two *Xiphister* congeners, the larger canine teeth on the lower jaw create two distinct clusters of residual stress, but are still within the bounds of functional homodonty.

Similar to the lower jaw, we observe in the upper jaw some individuals from each species with functionally heterodont teeth (Figure S2 and Table S4). However, we only observe these functionally heterodont teeth close to the jaw joint in the upper jaw. In the upper jaw, *P. chirus* and *A. purpurescens* have the highest proportion of functionally heterodont teeth with mean proportion of functionally heterodont teeth (12.9% for *P. chirus* and 9.6% for *A. purpurescens*), while both *Xiphister* species had lower mean proportions (3% for *X. atropurpureus* and 7.8% for *X. mucosus*). Average squared residuals follow similar patterns to the proportion of functionally heterodont teeth and can be found in the supporting material (Table S4).

### 3.2 Ontogenetic Trajectories of Focal Species

We next calculated ontogenetic trajectories for each jaw and dental trait for each species. All traits had at least one species that deviates from isometric growth (Figure 6 and Table 5, expanded in Table S2). For our measured jaw morphometrics, all species have positively allometric growth in the opening in‐lever of the lower jaw and premaxilla length. For the closing in‐lever of the lower jaw, lower jaw out‐lever, upper jaw closing in‐lever, only *X. mucosus* has isometric growth; all other species are positively allometric. For lower jaw width and upper jaw out‐lever, *X. mucosus* has positively allometric growth while the other three species are isometric. For premaxilla height, *A. purpurescens* has allometric growth and the other three species are isometric. For mean teeth height and width in the lower jaw, *A. purpurescens* and *P. chirus* are positively allometric, while both *Xiphister* species have isometric growth. For the mean height, width, and surface area of teeth in the upper jaw *A. purpurescens* has positively allometric growth, and the other three species are isometric.

### 3.3 How Diets Group in Multivariate Space

Each of our three LDAs separates carnivores from omnivores and herbivores in our focal species (Figure 5); however, each LDA groups herbivores and omnivores closer together, with overlap specifically between *X. atropurpureus* and *X. mucosus*. In our first LDA (LDA‐A, Figure 5A) we restricted our data to jaw morphometrics. LDA‐A separates carnivores from other groups but has large overlap between omnivores and herbivores. In LDA‐B (Figure 5B), we expanded our data to jaw morphometrics and dental characteristics. LDA‐B has increased separation from carnivores on LD1, and has increased separation between omnivores and herbivores, primarily on LD2. In LDA‐C (Figure 5C) we expanded our data by adding the proportion of functionally heterodont teeth in the lower and upper jaws to jaw morphometrics and dental characteristics. LDA‐C has increased separation between omnivores and herbivores, and greater separation still between those groups and carnivores. In each LDA, we observe a gradient within each diet group from smaller specimens (lighter) to larger specimens (darker), with smaller specimens generally closer to the center and large specimens generally having more extreme absolute values. This may be due to differentiation between and within diet groups over ontogeny.

## 4 Discussion and Conclusion

### 4.1 Different Morphotypes Across Diets

In our focal pricklebacks, diet demands fundamentally different solutions in the jaws and teeth. Carnivorous *A. purpurescens*, with slender jaws and many teeth, have a distinctly different feeding morphology from either omnivorous *P. chirus* and *X. atropurpureus* or herbivorous *X. mucosus*. This suggests that dedicated consumption of animal prey requires different functional morphology than any amount of plant matter consumption. The carnivorous *A. purpurescens* has proportionately larger jaws with villiform teeth for piercing into prey, while omnivores and herbivores (*P. chirus*, *X. atropurpureus*, and *X. mucosus*) have shorter jaws with fewer, wider teeth, more suited for grip and crushing force (Figures 4 and 5, Tables 2 and 3). In addition, *A. purpurescens*' relatively larger and wider jaws maximizes potential prey size (Kopf et al. 2021; Mittelbach and Persson 1998; Wainwright and Richard 1995), and their dental battery composed of numerous slender, pointed teeth, may improve gripping ability and reduce slippage when capturing soft prey (Carr et al. 2021; Cohen et al. 2020a; Galloway et al. 2016). In *A. purpurescens*, smaller teeth support larger teeth, spreading the relative impact of biting (Cohen et al. 2020a). This strategy, while ideal for soft prey, relies on many individually fragile teeth. In pricklebacks that consume plants, we observe conical and rounded teeth, able to withstand more force; at the cost of fewer individual points of contact with prey. Omnivores and herbivores possess more functionally heterodont teeth in the lower jaw, and this difference shows that eating plants requires more separation in roles between larger teeth and smaller teeth. These functionally heterodont teeth are at the front of the jaw, aiding with prey capture, while high‐stress teeth at the rear of the jaw are poised for applying force (Figure S1). In addition to biting, pricklebacks also implement spin feeding, the process of tearing pieces off of a larger prey item through long‐axis body rotation, and their arrangement of conical teeth may provide the balance of strength and grip necessary for feeding on plants in this fashion (Crawford et al. 2025; Horn and Ojeda 1998; Miller and Marshall 1987; Yoshiyama et al. 1996).

Foraging for plant material is a derived phenotype in fishes, possibly an evolutionary “dead end” due to the necessary adaptations (Egan et al. 2018 *; but see* Kolmann et al. 2024). We hypothesized that the herbivores would be the most different from the carnivores, similar in scale to digestive adaptations seen in herbivorous pricklebacks, with omnivores possessing an intermediate morphology. Instead, we found that herbivores and omnivores both have fairly different feeding morphologies compared to carnivores (Figure 5). The top traits separating omnivores from herbivores are related to the strength of jaw closing and dental characteristics like tooth width and surface area (Table 4). While no individual traits are significantly different in means between *Xiphister* species, there is separation in multivariate space, with the overlap between omnivorous and herbivorous groups being composed of juvenile *Xiphister* specimens (Figure 5, Tables 3 and 4).

**Table 4:** Linear Discriminant Analysis Loadings for Figure 5.

Trait	LDA 1		LDA 2		LDA 3	
	LD1	LD2	LD1	LD2	LD1	LD2
LJ out‐lever	**−2.45**	**−1.67**	−0.99	**−2.1**	−1.74	−0.31
LJ closing in‐lever	**1.31**	**0.92**	0.81	1.46	0.78	0.95
LJ opening in‐lever	0.65	0.26	0.81	0.6	0.64	−0.03
Jaw width	−0.74	−0.15	−1.14	0.02	−0.71	0.02
Pmax height	0.49	0.32	0.88	0.29	0.6	−0.01
Pmax length	−0.14	−0.41	−0.45	−0.08	0.14	−0.35
UJ closing in‐lever	**−1.26**	**1.21**	−2.85	0.27	**−2.38**	−0.47
UJ out‐lever	−1.01	0.5	−1.67	0.01	−0.88	−0.32
LJ teeth mean height			2.17	−1.1	1.36	**−3.02**
UJ teeth mean height			**−2.93**	1.19	−2.26	2.69
LJ teeth mean width			1.63	0.57	−0.05	0.98
UJ teeth mean width			2.24	**−5.11**	2.38	**−5.03**
LJ teeth mean SA			**−6.92**	0.86	**−3.47**	**3.65**
UJ teeth mean SA			**4.74**	**2.38**	**2.94**	−0.07
LJ teeth number			−1.19	−0.77	−1.38	−0.4
UJ teeth number			1.28	0.24	1.39	0.5
LJ proportion heterodont					0.12	0.58
UJ proportion heterodont					−0.64	−0.06

*Note:* Top three loadings for LD1 and LD2 in bold.

Lastly, we find that the omnivorous *P. chirus* is overall the most different from the carnivorous *A. purpurescens*, with relatively shorter jaws and fewer, much larger, more rounded teeth (Figure 4, Tables 2 and 3). Despite *P. chirus* and *X. atropurpureus* both being omnivorous, the more robust teeth of *P. chirus* may reflect selection for prey items that are materially harder to process. A rounded tooth morphology is better suited for absorbing the force necessary to fracture hard prey items like crustaceans (Crofts et al. 2020; Crofts and Summers 2014; Deng et al. 2022), which matches increased chitinase enzyme expression for digesting crustaceans found in the gut of *P. chirus* in previous studies (German et al. 2014; Herrera et al. 2022).

### 4.2 Divergent Ontogenetic Scaling Trajectories

We confirmed that different diets require different scaling of feeding morphologies in our focal pricklebacks; however, these trends are opposed to established allometric trends in the digestive system. These divergent ontogenetic scaling trajectories between pricklebacks of different diets may demonstrate the relative specialization of the oral jaws and dentition versus the digestive system for each diet. Previous research in pricklebacks has revealed ontogenetic changes in digestive morphology and physiology in herbivores and omnivores, such as positively allometric gut lengths and changes in enzyme expression for processing plants (German et al. 2004, 2014, 2015; German and Horn 2006; Heras et al. 2020; Herrera et al. 2022). However, we found the opposite pattern in feeding morphology, with many traits that scale with positive allometry in our carnivore, fewer instances of allometry in omnivores, and mostly isometry in our herbivore (Figure 6, Table 5 and Table S2). Carnivorous fishes often benefit from positively allometry in overall jaw size, as it allows for relatively larger prey to be tackled and pierced as jaw muscle size improves (Davis et al. 2011; Mittelbach and Persson 1998; Wainwright and Richard 1995). The herbivore *X. mucosus* is largely isometric feeding morphology, and this may mean that they interact with plants in consistent ways over ontogeny. One of the few traits with positive allometry in *X. mucosus* is jaw width, which may allow for relatively larger prey items to be consumed over ontogeny compared to the pricklebacks with isometric jaw width. This is likely important as *X. mucosus* is intake limited, which means they maximize the amount of algae they consume for higher energy yield, and have positive allometry of gut length, allowing them to process proportionally more prey with growth (German et al. 2015; Rankins et al. 2023). *X. mucosus* also grows larger than other the focal species, further adding evidence towards herbivory necessitating larger body size (Kells et al. 2016; Mecklenburg et al. 2002). The two *Xiphister* sister species have very similar feeding morphology, in spite of dietary differences. However, these species often differ in the scaling relationships of feeding morphology traits. These scaling relationships may lay the foundation for divergence in larger adult fish outside our sample sizes.

**Table 5:** Ontogenetic regressions for traits that contribute the most to differences between diet groups.

Trait	Species	Rsquared	intercept	Intercept 95% CI	Slope	Slope 95% CI	Expected Isometric Slope	Allometry
LJ Out‐lever	*Anoplarchus purpurescens*	0.99	−1.35	−1.678, −1.047	1.41	1.273, 1.558	1	P
LJ Out‐lever	*Phytichthys chirus*	1	−1.22	−1.392, −1.067	1.19	1.124, 1.267	1	P
LJ Out‐lever	*Xiphister atropurpureus*	0.99	−0.95	−1.176, −0.745	1.12	1.037, 1.22	1	P
LJ Out‐lever	*Xiphister mucosus*	0.99	−0.72	−0.926, −0.534	1.02	0.939, 1.106	1	I
LJ Closing IL	*Anoplarchus purpurescens*	0.97	−2.3	−2.799, −1.879	1.32	1.133, 1.548	1	P
LJ Closing IL	*Phytichthys chirus*	0.98	−2.05	−2.432, −1.718	1.16	1.012, 1.324	1	P
LJ Closing IL	*Xiphister atropurpureus*	0.98	−2.3	−2.675, −1.969	1.3	1.158, 1.459	1	P
LJ Closing IL	*Xiphister mucosus*	0.97	−1.87	−2.29, −1.502	1.08	0.927, 1.262	1	I
UJ Closing IL	*Anoplarchus purpurescens*	0.95	−2.01	−2.786, −1.372	1.56	1.27, 1.908	1	P
UJ Closing IL	*Phytichthys chirus*	0.99	−1.54	−1.826, −1.285	1.23	1.121, 1.358	1	P
UJ Closing IL	*Xiphister atropurpureus*	1	−1.43	−1.596, −1.274	1.19	1.124, 1.261	1	P
UJ Closing IL	*Xiphister mucosus*	0.9	−1.31	−2.168, −0.66	1.13	0.853, 1.496	1	I
LJ teeth mean height	*Anoplarchus purpurescens*	0.86	−4.61	−5.934, −3.653	1.56	1.124, 2.154	1	P
LJ teeth mean height	*Phytichthys chirus*	0.95	−3.94	−4.567, −3.43	1.3	1.071, 1.568	1	P
LJ teeth mean height	*Xiphister atropurpureus*	0.87	−4.24	−5.41, −3.382	1.33	0.968, 1.831	1	I
LJ teeth mean height	*Xiphister mucosus*	0.83	−3.23	−4.136, −2.603	0.9	0.626, 1.279	1	I
UJ teeth mean height	*Anoplarchus purpurescens*	0.95	−4.69	−5.421, −4.099	1.45	1.18, 1.777	1	P
UJ teeth mean height	*Phytichthys chirus*	0.95	−3.85	−4.431, −3.38	1.13	0.922, 1.382	1	I
UJ teeth mean height	*Xiphister atropurpureus*	0.88	−3.95	−4.848, −3.289	1.09	0.809, 1.473	1	I
UJ teeth mean height	*Xiphister mucosus*	0.78	−3.46	−4.491, −2.765	0.88	0.59, 1.326	1	I
UJ teeth mean width	*Anoplarchus purpurescens*	0.95	−5.26	−5.949, −4.701	1.35	1.098, 1.661	1	P
UJ teeth mean width	*Phytichthys chirus*	0.94	−4.48	−5.151, −3.936	1.21	0.97, 1.502	1	I
UJ teeth mean width	*Xiphister atropurpureus*	0.91	−4.51	−5.305, −3.897	1.14	0.882, 1.481	1	I
UJ teeth mean width	*Xiphister mucosus*	0.87	−4.08	−4.939, −3.458	0.96	0.698, 1.328	1	I
LJ teeth mean SA	*Anoplarchus purpurescens*	0.9	−9.64	−11.86, −7.967	3.03	2.277, 4.035	2	P
LJ teeth mean SA	*Phytichthys chirus*	0.98	−8.17	−9.074, −7.375	2.7	2.354, 3.098	2	P
LJ teeth mean SA	*Xiphister atropurpureus*	0.9	−8.72	−10.815, −7.136	2.72	2.048, 3.614	2	P
LJ teeth mean SA	*Xiphister mucosus*	0.88	−6.65	−8.16, −5.533	1.8	1.33, 2.449	2	I
UJ teeth mean SA	*Anoplarchus purpurescens*	0.95	−9.93	−11.362, −8.753	2.86	2.334, 3.511	2	P
UJ teeth mean SA	*Phytichthys chirus*	0.95	−8.13	−9.368, −7.127	2.43	1.99, 2.971	2	I
UJ teeth mean SA	*Xiphister atropurpureus*	0.91	−8.54	−10.295, −7.197	2.41	1.842, 3.161	2	I
UJ teeth mean SA	*Xiphister mucosus*	0.88	−7.19	−8.761, −6.046	1.83	1.343, 2.5	2	I

### 4.3 Outgroup Perspective: Diverse Feeding Traits Within Each Diet

Lastly, we found there are no clear “herbivore”, “omnivore”, or “carnivore” phenotypes in our outgroup species. These three species span across the superfamily Zoarcoidea and represent another instance of each of our dietary categories (herbivorous, omnivorous, and carnivorous; Figures 1 and 3). In our focal species, we found that diet groups have differences in functional morphology, and diverge via the scaling of traits over ontogeny (Figures 4, 5, and 6, Tables 2, 3, 4, 5). However, when we compared our focal fishes to outgroup species with known diets (Figures 1 and 3), we find dissimilar morphologies within each dietary category. Our first outgroup, the carnivore *P. laeta*, has relatively short jaws and a small number of large blunted teeth, similar to *P. chirus* and *Xiphister*. Our second outgroup, the herbivore *C. violaceus*, has a similar jaw morphology and dentition to our focal carnivore *A. purpurescens*, with large jaws and a high number of pointed teeth that vary in size. Lastly, the omnivore *L. sagitta* has relatively smaller, pointed teeth with a high number of teeth on the upper jaw and a much lower number in the lower jaw, a unique phenotype compared to our other species. There are many potential reasons for the outgroups fishes not following the pattern of our focal species, such as strong phylogenetic inertia, where individual species are limited and shaped by their shared ancestry moreso than dietary nuances; or possibly differences in specific feeding behavior and interaction with prey material properties within each dietary group. Within the pricklebacks, the ability to assimilate plants has arisen at least two times (Kim et al. 2014). As convergent evolutions, *C. violaceus* has been described as using a different type of herbivory than *X. mucosus*, relying on microbial fermentation to break down prey items instead of bulk intake (German et al. 2015). The carnivore *P. laeta* preys largely on crustaceans and other invertebrates, selecting for fewer, blunted teeth, similar to *P. chirus* (Hughes 1985; Peden and Hughes 1984). Lastly, *L. sagitta* is an omnivore with a functionally carnivorous gut, consuming a mix of plant and animal material, but does not effectively digest plants (Rankins et al. 2023). This species does not conform to any of our previous morphotypes, and the large differences in tooth number may indicate unique functional differences between the upper and lower jaws.

What a fish consumes is based on what prey are available in the habitat, what it can recognize and capture (morphology, performance, behavior), and what it can assimilate (nutritional physiology), among myriad other factors (Wainwright and Bellwood 2002; Wainwright and Richard 1995). Pricklebacks possess diverse oral jaws and feed upon diverse prey types, but there are no unifying morphological trends across the larger family that distinguish diet categories (i.e., carnivores, omnivores, herbivores). Drawing from other instances of dietary specializations in teleosts reveals even more diverse feeding morphologies for different plant prey types. In the speciose East African Lake cichlids, herbivorous clades have partitioned the seemingly straightforward niche of eating epilithic algae into several distinct functional groups: grazers, (which can be further divided into scrapers, combers, and suckers), browsers, and peckers (Yamaoka 1987, 1997; Tada et al. 2017). Each functional group has unique jaw and tooth morphologies as well as distinct feeding behaviors for procuring algae from the rocks. In pacus and piranhas (serrasalmidae), we see differential performance of dentition based on prey type. Blunt‐toothed frugivorous pacus like *Colossoma* undergo high work to crush fruit, while carnivorous piranhas use their sharp, bladed dentition slice through flesh with low work (Rosen et al. 2025). Herbivorous parrotfishes are divided into two groups, grazers and scrapers, with differences in beak shape, jaw muscle size, and feeding behavior (Bellwood et al. 2015). However, eating plants in fishes does not necessarily require large specializations to the oral jaws. Several studies have found morphology to track more closely to feeding behavior or microhabitat rather than diet type (Douglas and Matthews 1992; Grossman 1986; Motta et al. 1995). More research on the musculature of the jaws as well as the specific feeding behaviors involved in prey capture and prey properties in the pricklebacks may help clarify how their diverse morphologies arise (Motta et al. 1995; Norton 1995; Roberts et al. 2018). For example, the relative configuration and allometry of muscles strongly influences jaw function, as muscle force scales with cross‐sectional area (Norton and Cook 1998; Velasco‐Hogan and Meyers 2021). Additionally, while prey acquisition occurs primarily via the oral jaws and dentition, the majority of prey processing often occurs in the pharyngeal jaws in fishes (Lauder 1983; Wainwright 2005). Prey processing in the pharyngeal jaws is influenced by prey material properties, especially plants or hard‐shelled prey that require mechanical processing for effective digestive assimilation (Burress 2016; Gidmark et al. 2015; Tada et al. 2017). There are many different avenues to continue to explore the relationship between dietary diversity, nutritional physiology, and functional morphology using the pricklebacks.

## Author Contributions

**R. C. Hoover:** conceptualization, methodology, investigation, software, visualization, writing – original draft, writing – review and editing. **Kassandra L. Ford:** writing – original draft, funding acquisition, supervision, writing – review and editing. **Karly E. Cohen:** conceptualization, software, writing – original draft, writing – review and editing. **Cassandra M. Donatelli:** conceptualization, software, writing – original draft, writing – review and editing, funding acquisition.

## Conflicts of Interest

The authors declare no conflicts of interest.

## Supporting information

Supporting File 1

Supporting File 2

Supporting File 3

Supporting File 4

Supporting File 5

Supporting File 6

Supporting File 7

Supporting File 8

## Data Availability

All microCT scans are freely available on morphosource.org.
