# Supplementary figures and images for: Functional Morphology of the Oral Jaws and Dentition Across Diverse Diets and Ontogeny in Prickleback Fishes (Stichaeidae)
Source: J Morphol. 2026 May 12;287:e70131. doi: 10.1002/jmor.70131 (PMC13162304; doi:10.1002/jmor.70131)

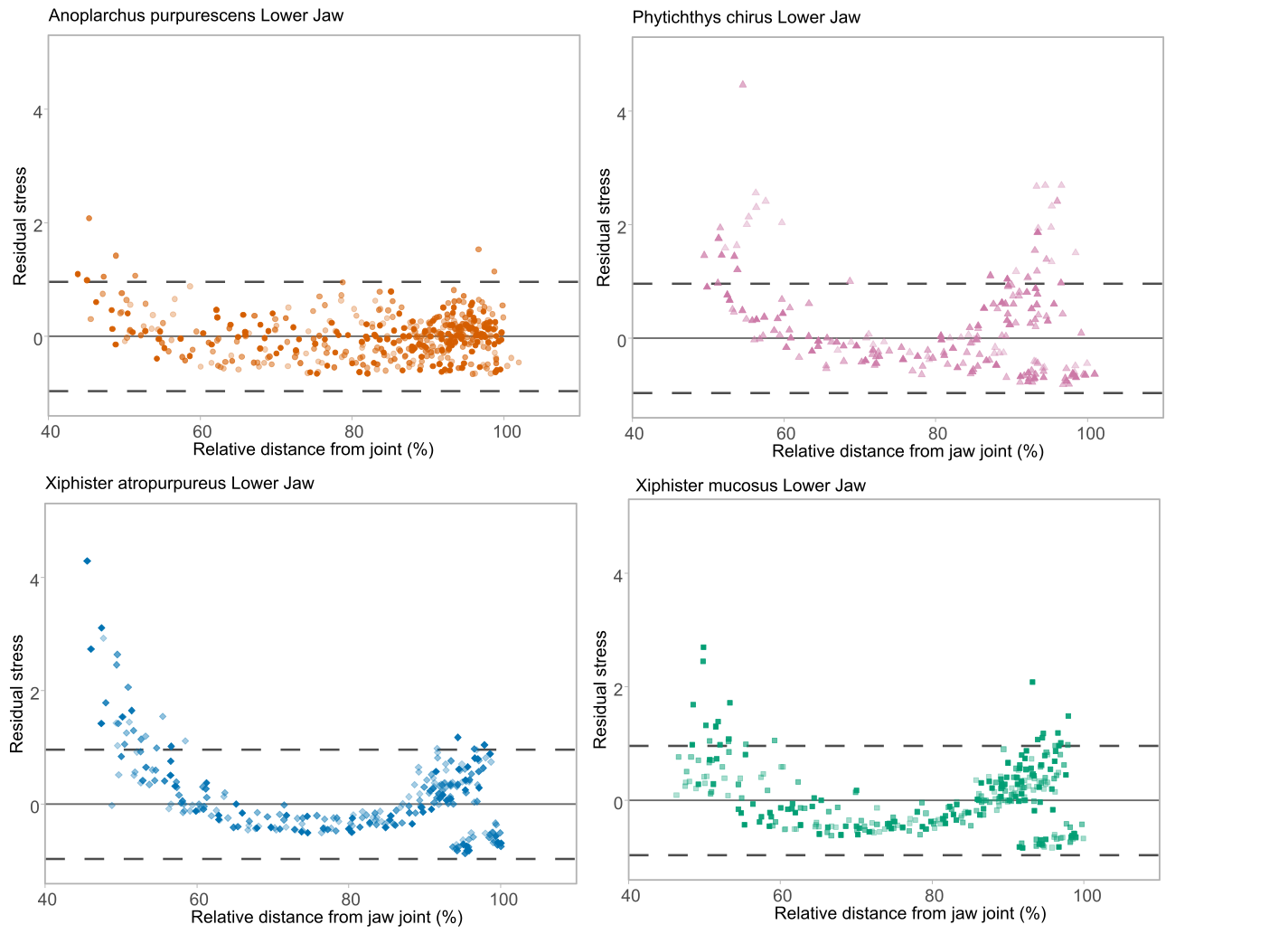

Supplement: Supplementary file 1 — Supporting File 1 [file JMOR-287-e70131-s005.tiff]

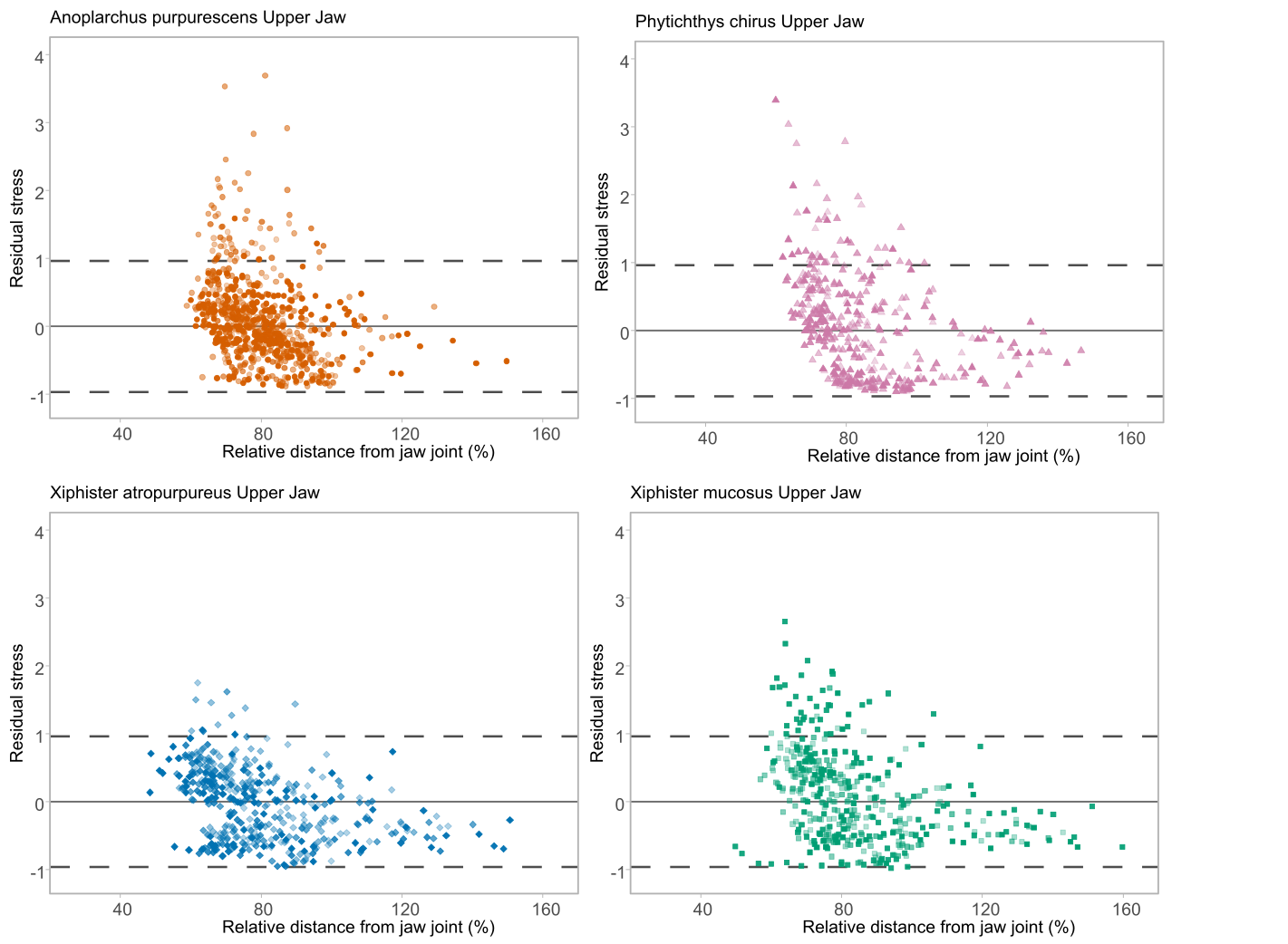

Supplement: Supplementary file 2 — Supporting File 2 [file JMOR-287-e70131-s001.tiff]
